# Accuracy of rapid diagnosis of *Talaromyces marneffei*: A systematic review and meta-analysis

**DOI:** 10.1371/journal.pone.0195569

**Published:** 2018-04-05

**Authors:** Chuanyi Ning, Jingzhen Lai, Wudi Wei, Bo Zhou, Jiegang Huang, Junjun Jiang, Bingyu Liang, Yanyan Liao, Ning Zang, Cunwei Cao, Hui Chen, Li Ye, Hao Liang

**Affiliations:** 1 Life Sciences Institute, Guangxi Medical University, Nanning, Guangxi, China; 2 Guangxi Key Laboratory of AIDS Prevention and Treatment, Guangxi Medical University, Nanning, Guangxi, China; 3 Bio-safety Level-3 Laboratory, Guangxi Medical University, Nanning, Guangxi, China; 4 School of Public Health, Guangxi Medical University, Nanning, Guangxi, China; 5 The First Affiliated Hospital, Guangxi Medical University, Nanning, Guangxi, China; Zhejiang University, CHINA

## Abstract

**Background:**

To examine the accuracy of Rapid Diagnosis of *Talaromyces marneffei* (RDTM) in order to improve diagnosis and treatment for clinical measures and reduce the mortality due to associated infections.

**Methods:**

In this systematic review and meta-analysis, we screened PubMed, Ovid (Cochrane library) and Web of Science, Chinese database CNKI and Wanfang for articles published between 1956 and December, 2017. Data were taken from cross-sectional studies as well as from baseline measurements in longitudinal studies with clinical follow-up. Articles were excluded if they did not contain a cohort with *T*. *marneffei* and a control cohort or a cohort with standard fungus culture. Data were extracted by two authors and checked by three for accuracy. For quality assessment, modified QUADAS-2 criteria were used.

**Results:**

The 26 included diagnostic studies enrolled 5,594 objectives in 632 patients with *T*. *marneffei* infections and 2,612 negative controls between 1996 and 2017 in Thailand, Vietnam and China. The total combined sensitivity and specificity of rapid diagnosis of *T*. *marneffei* was 0.82 (95% CI: 0.68–0.90) and 0.99 (95% CI: 0.98–1.00). According to the experimental method, the included studies can be divided into three subgroups, including PCR-based, ELISA-based and others. The results showed these three subgroups had a highly pooled specificity of 1.00 (95% CI: 0.99–1.00), 0.99 (0.98–1.00) and 0.97 (95% CI: 0.91–1.00), respectively, while combined sensitivity was 0.84 (95% CI: 0.37–0.98), 0.82 (95% CI: 0.64–0.92) and 0.77 (95% CI: 0.54–0.91), respectively.

**Conclusions:**

Although serological methods with a high specificity is essential for potential rapid diagnostic, false-negative results can be obtained in the serum samples, there is no suitable rapid serological test to refer to as is the case with TM infection.

## Introduction

*Talaromyces marneffei* (TM), formerly named *Penicillium marneffei* (also called PSM, or P.M) [1, 2], was first discovered in 1956 from the hepatic lesions of a bamboo rat (*Rhizomys sinensis*) which dying of disseminated mycosis that had been maintained in captivity for experimental infections at the Pasteur Institute of South Vietnam [3]. *T*. *marneffei* is endemic to Southeast Asia and South China as an opportunistic infectious disease in immunocompromised individuals [4]. The incidence of systemic *T*. *marneffei* infection has grown up rapidly in recent years, in consistent with the increasing incidence of HIV infections [5]. In endemic areas, for instance northern Thailand, it has become the third most common opportunistic infection in patients with AIDS, after tuberculosis and cryptococcosis [6]. The proportion of HIV patients infected with *T*. *marneffei* currently is 30% in Thailand, and ~10% in southern China [4, 5]. In addition, in these areas, about 50,000 HIV positive patients are newly infected by *T*. *marneffei* each year, leading to an annual death rate of up to 10% [7]. *T*. *marneffei* infected AIDS present serious systemic disease with fever, anemia, weight loss, skin lesion, respiratory signs, generalized lymphadenopathy, and hepatosplenomegaly. Clinical experiences show that treatment using amphotericin B, itraconazole, ketoconazole, and fluconazole alone or combined appropriately during the early stage can effectively combat T. *marneffei* [8, 9].

At present, late diagnosis is the main reason for the high fatality in TM patients co-infected with HIV, combining other underlying diseases or simplex *T*. *marneffei* [10, 11]. *T*. *marneffei* is usually diagnosed by microscopic identification of the fungus in clinical specimens and by cultivation techniques, based on its characteristic morphological and dimorphic properties, which grow as a mycelium form at 25°C and as a yeast form at 37°C [12, 13]. However, such procedures are relatively time-consuming, requiring about ten days, which might negatively affect the selection of an appropriate therapy. Clinical specimens for culture that are commonly used include bone marrow aspirate (100%), skin biopsies, skin scrapings (90%), blood (76%), sputum, urine and stool analysis, lymph node, liver and bronchoalveolar lavage pellet, pleural and cerebrospinal fluid analysis, pharyngeal ulcer and palatal papule scrapings and kidney, pericardium, stomach or intestine analysis [14]. Although fungi culture is featured with high accuracy of diagnosis and wide applicability of various specimens, being time-consuming has pulling these advantages back. Therefore, rapid diagnosis of T. marneffei is in urgent need for its rapid turnaround time, high accuracy, cost saving, and potential to reduce fatality. Currently, there is a significant emphasis on rapid diagnosis methods due to their high sensitivity and specificity, including polymerase chain reaction (PCR) [15–17], mainly real-time quantification PCR [18, 19], nested PCR [20–23], in situ hybridization PCR [24–26], enzyme-linked immunosorbent assay (ELISA) [27–35] and biochemical criterion tests [36–45].

However, in light of a reported sensitivity ranging from 10 to 100%, whereas specificity usually exceeds 95%, it is unclear which rapid diagnosis technique is preferable. The aim of this review is to investigate whether RDTM is sufficiently specific or sensitive in order to enable early treatment. This study has the potential to provide a rapid diagnostic method for *T*. *marneffei* infection, thus enabling early therapeutic management.

## Materials and methods

### Search strategy and study selection

We searched PubMed, Ovid (Cochrane library), and Web of Science, and WanFang (a Chinese bibliographic database) and CNKI (China National Knowledge Infrastructure) for published articles which reported methods for detecting *Talaromyces marneffei between 1956 and December*, *2017*. The systematic literature search syntaxs were ("Talaromyces marneffei"[Text Word] OR "Penicillium marneffei"[Text Word] OR "P. marneffei"[Text Word] OR "T. marneffei"[Text Word]) AND ((“diagnosis"[MeSH Terms] OR diagnostic[Text Word] OR detect[Text Word] OR detection[Text Word]) OR (“sensitivity and specificity"[MeSH Terms] OR sensitivity [Text Word] OR specificity [Text Word]) OR “ROC Curve”[MeSH Terms]) AND ("1956/01/01"[PubDate]: "2017/12/31"[PubDate]).

A study was included in the meta-analysis when it met all following criteria: (1) a study for diagnosis of *Talaromyces marneffei*; (2) use of the reference standards; (3) use of a control group; (4) provision of true positive, false positive, true negative and false negative directly or indirectly.

### Data extraction and quality assessment

Two trained reviewers independently screened the articles and judged research eligibility; disagreements were eliminated through team discussion. Data retrieved from the articles included publication year, country, participants, specimen resource, study design, RDTM method (PCR, ELISA, LA or other rapid serodiagnosis methods), blinding, true positive, false positive, true negative, false negative, sensitivity and specificity data, positive and negative predictive values. Table 1 shows the details of the extracted data. We assessed the methodological quality of each article on case selection, study design, golden standard diagnosis, blinding and so on, according to the Quality Assessment of Diagnostic Accuracy Studies tool (QUADAS2)[46].

**Table 1:** Characteristics of included studies (Ordered by the subgroup).

Study ID	Countries	Participants	Specimen resource	Study design	Test methods	Blinding	Positive/negative predictive value % (95% CI)
Chen 2011a [17]	China	Cases: 21 Controls: 12	Bone marrow culture	Case series with culture proven *T*. *marneffei* infection	PCR, ITS1 and ITS4 DNA	Unclear	PPV: 100.0 (73.5, 100.0) NPV: 100.0 (83.8, 100.0)
Chen 2011b [17]	China	Cases: 21 Controls: 12	Blood culture	Case series with culture proven *T*. *marneffei* infection	PCR, ITS1 and ITS4 DNA	Unclear	PPV: 100.0 (73.5, 100.0) NPV: 100.0 (83.8, 100.0)
Chen 2011c [17]	China	Cases: 21 Controls: 12	Blood	Case series with culture proven *T*. *marneffei* infection	PCR, ITS1 and ITS4 DNA	Unclear	PPV: 100.0 (73.5, 100.0) NPV: 100.0 (83.8, 100.0)
Hien 2016a [19]	Vietnam	Cases: 27 Controls: 20	Plasma	Case series with culture proven *T*. *marneffei* infection	PCR, MP1 gene	Unclear	PPV: 100.0 (82.4, 100.0) NPV: 71.4 (51.3, 86.8)
Hien 2016b [19]	Vietnam	Cases: 23 Controls: 20	Plasma	Case series with culture proven *T*. *marneffei* infection	PCR, MP1 gene	Unclear	PPV: 100.0 (73.5, 100.0) NPV: 64.5 (45.4, 80.8)
Pongpom 2009 [20]	Thailand	Cases: 35 Controls: 365	Serum	Case series with culture proven *T*. *marneffei* infection	Nested PCR, 18S ribosomal DNA	Unclear	PPV: 100.0 (85.8, 100.0) NPV: 97.1 (94.8, 98.5)
Prariyachatigul 2003 [22]	Thailand	Cases: 2 Controls: 17	Blood	Case series with culture proven *T*. *marneffei* infection (cases were unknown when operate the PCR assay)	Semi nested PCR, 18S ribosomal RNA	Yes	PPV: 100.0 (15.8, 100.0) NPV: 100.0 (80.5, 100.0)
Desakorn 2002a [31]	Thailand	Cases: 37 Controls: 300	Urine	Case series with culture proven *T*. *marneffei* infection	ELISA, IgG for the detection of *T*. *marneffei* urinary antigen	Unclear	PPV: 81.4 (66.6, 91.6) NPV: 99.3(97.5, 99.9)
Desakorn 2002b [31]	Thailand	Cases: 37 Controls: 300	Urine	Case series with culture proven *T*. *marneffei* infection	ELISA, IgG for the detection of *T*. *marneffei* urinary antigen	Unclear	PPV: 85.7 (71.5, 94.6) NPV: 99.7(98.2, 100.0)
He 2016 [34]	China	Cases: 115 Controls: 277	Serum	Case series with culture proven *T*. *marneffei* infection	ELISA	Unclear	PPV: 59.1 (50.9, 66.9) NPV: 89.9 (85.3, 93.4)
Kaufman 1996b [32]	Thailand	Cases: 17 Controls: 15	Serum	Case series with culture proven *T*. *marneffei* infection	ELISA	Unclear	PPV: 100.0 (69.2, 100.0) NPV: 68.2 (45.1, 86.1)
Panichakul 2002a [47]	Thailand	Cases: 18 Controls: 148	Serum at a dilution of 1:2	Case series with culture proven *T*. *marneffei* infection	ELISA, with MAb 8C3 for the detection of *T*. *marneffei* antigen	Unclear	PPV: 100.0 (75.3, 100.0) NPV: 96.7 (92.5, 98.9)
Panichakul 2002b [47]	Thailand	Cases: 18 Controls: 148	Undiluted serum	Case series with culture proven *T*. *marneffei* infection	ELISA, with MAb 8C3 for the detection of *T*. *marneffei* antigen	Unclear	PPV: 100.0 (81.5, 100.0) NPV: 98.7 (95.3, 99.8)
Prakit 2016 [28]	Thailand	Cases: 45 Controls: 232	Serum	Case series with culture proven *T*. *marneffei* infection	ELISA, using MAb 4D1 for detecting Penicillium marneffei antigen	Unclear	PPV: 100.0 (92.1, 100.0) NPV: 100.0 (98.4, 100.0)
Sansanee 2003 [29]	Thailand	Cases: 53 Controls: 240	Serum	Case series with culture proven *T*. *marneffei* infection	ELISA,which employs 8B11 and 8C3 to detect *T*. *marneffei* antigens	Unclear	PPV: 89.1 (77.8, 95.9) NPV: 98.3 (95.8, 99.5)
Wang 2011a [35]	China	Cases: 20 Controls: 540	Serum	Case series with culture proven *T*. *marneffei* infection	ELISA,with MAb-Mab Mp1p for detecting *T*. *marneffei* antigen	Unclear	PPV: 84.6 (54.6, 98.1) NPV: 98.4 (96.9, 99.2)
Wang 2011b [35]	China	Cases: 20 Controls: 540	Serum	Case series with culture proven *T*. *marneffei* infection	ELISA,with PAbs-Mab Mp1p for detecting *T*. *marneffei* antigen	Unclear	PPV: 83.3 (58.6, 96.4) NPV: 99.1 (97.9, 99.7)
Wang 2011c [35]	China	Cases: 20 Controls: 540	Serum	Case series with culture proven *T*. *marneffei* infection	ELISA, with Mp1p IgG for detecting *T*. *marneffei* antigen	Unclear	PPV: 42.9 (17.7, 71.1) NPV: 97.4 (95.7, 98.6)
Wang 2013a [27]	China	Cases: 15 Controls: 121	Serum	Case series with culture proven *T*. *marneffei* infection	ELISA, with Mp1p for detecting the antibody of *T*. *marneffei*	Unclear	PPV: 100.0 (15.8, 100.0) NPV: 90.3 (84.0, 94.7)
Wang 2013b [27]	China	Cases: 15 Controls: 121	Serum	Case series with culture proven *T*. *marneffei* infection	ELISA, with Mp1p for detecting the antigen of *T*. *marneffei*	Unclear	PPV: 100.0 (76.8, 100.0) NPV: 97.6 (93.1, 99.5)
Wang 2013c [27]	China	Cases: 15 Controls: 121	Serum	Case series with culture proven *T*. *marneffei* infection	ELISA,plus Double antibody sandwich ELISA	Unclear	PPV: 100.0 (73.5, 100.0) NPV: 99.2 (95.5, 100.0)
Desakorn 2002c [31]	Thailand	Cases: 37 Controls: 300	Urine	Case series with culture proven *T*. *marneffei* infection	Latex agglutination test, IgG for the detection of *T*. *marneffei* urinary antigen	Unclear	PPV: 94.9 (82.7, 99.4) NPV: 100.0 (98.8, 100.0)
Hu 2015 [45]	China	Cases: 82 Controls: 92	Serum	Case series with culture proven *T*. *marneffei* infection	G test, cut-off 75pg/ml	Unclear	PPV: 81.3 (70.7, 89.4) NPV: 78.8 (69.4, 86.4)
Kaufman 1996a [32]	Thailand	Cases: 17 Controls: 15	Serum	Case series with culture proven *T*. *marneffei* infection	Latex agglutination (LA) for antigen	Unclear	PPV: 100.0 (75.3, 100.0) NPV: 78.9 (54.4, 73.9)
Wei 2013 [43]	China	Cases: 74 Controls: 183	Blood and Bone marrow	Case series with culture proven *T*. *marneffei* infection (cases were unknown when operating tests)	Giemsa staining	Yes	PPV: 91.7 (77.5, 98.2) NPV: 81.4 (75.7, 86.3)
Zhou 2015 [44]	China	Cases: 48 Controls: 50	Serum	Case series with culture proven *T*. *marneffei* infection	Spectrophotometric detection of two Serums adenosine deaminase levels, cut-off	Unclear	PPV: 90.2 (76.9, 97.3) NPV: 80.7 (68.1, 90.0)

Note: PPV is Positive predictive value; NPV is Negative predictive value. The order of the included studies are showed by the subgroup analysis, the gray background are the molecular diagnostic group and Others group, the middle one is (no background) the ELISA group. The studies in each subgroup are ordered by alphabet of the first author.

### Data collection and analysis

Data were extracted either on article or study level when possible to reconstruct the 2 × 2 tables which were used to calculate sensitivity and specificity. The studies included were grouped by type of RDTM. For meta-analyzed the accuracy of RDTM, we selected to perform bivariate random-effects regression models, as recommended by the Cochrane Diagnostic Test Accuracy Working Group for which I^2^>50%, and combined the sensitivity and specificity estimates. Publication bias was investigated with visual inspection of funnel plots. The bivariate random-effects model to estimate takes into consideration the potential trade-off between sensitivity and specificity by explicitly incorporating this negative correlation in the analysis. Positive and negative of predictive values were computed and diagnostic likelihood ratios (DLR) were directly generated from pooled pair sensitivity and specificity estimates. The data was also used to plot summary receiver-operating characteristic (SROC) curves by establish the true positivity and false positivity (1—specificity) of each study. The closer the curve is to the upper left-hand corner, with the exact area under the curve (AUC) of the SROC curve plot, the better the overall accuracy of the test. Summary sensitivity and specificity estimates for each variate and subgroup were generated, along with 95% CIs (confidence intervals). All analyses were conducted using Stata 12.0 and Revman 5.0.

## Results

### Study selection and characteristic of included studies

**Fig 1:**
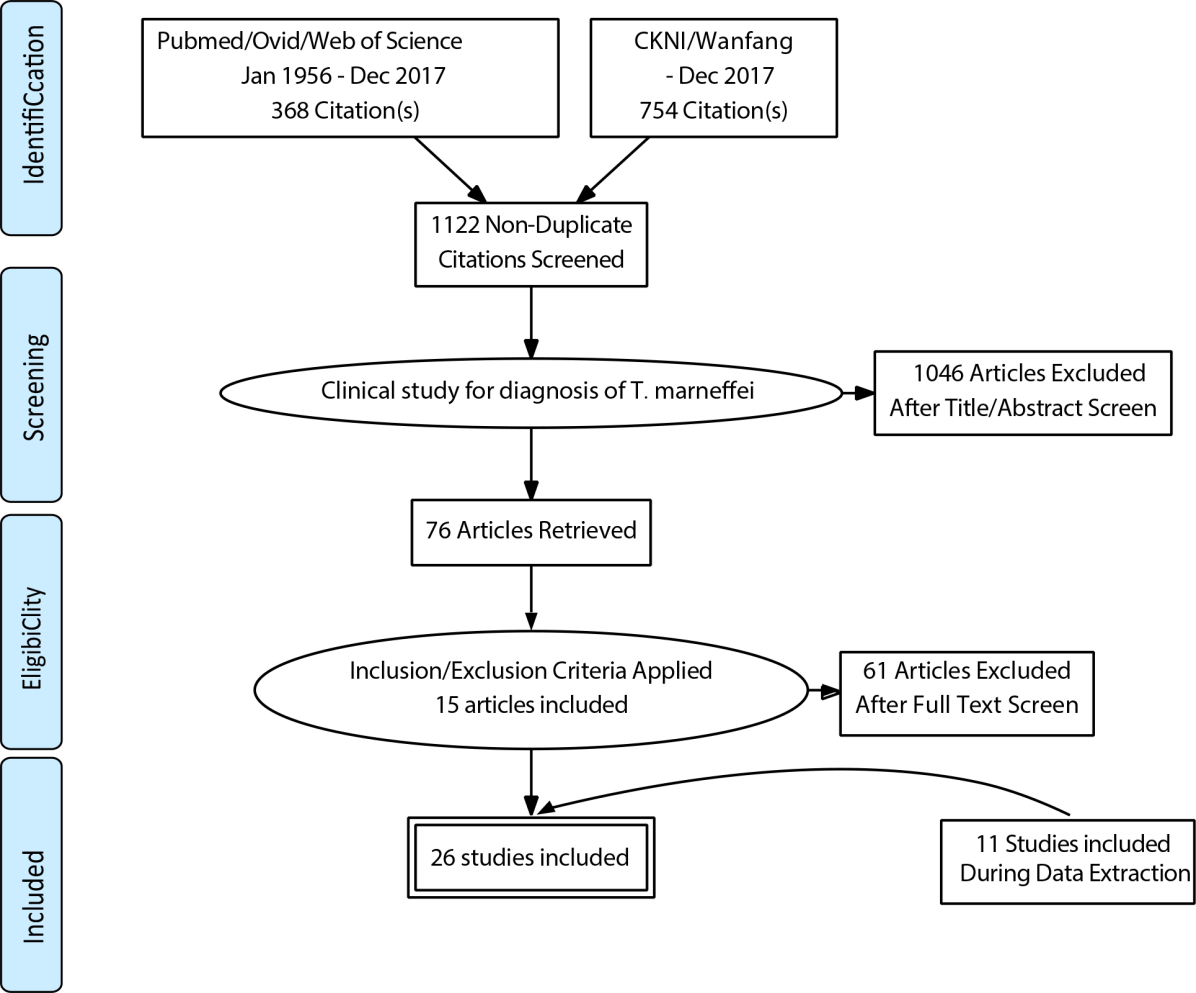
PRISMA (2009) flow diagram.

We identified 368 records from PubMed/Ovid/Web of Science and 510 and 244 articles from CNKI and Wanfang, respectively. If these articles, 1046 were excluded because their research contents did not comply with our criteria. We excluded another 61 articles because of specificity/sensitivity was absence or dual publication. Finally, due to some articles evaluated more than one RDTM, 15 full-text articles, including 26 studies were available for the meta-analysis (Fig 1).

**Fig 2:**
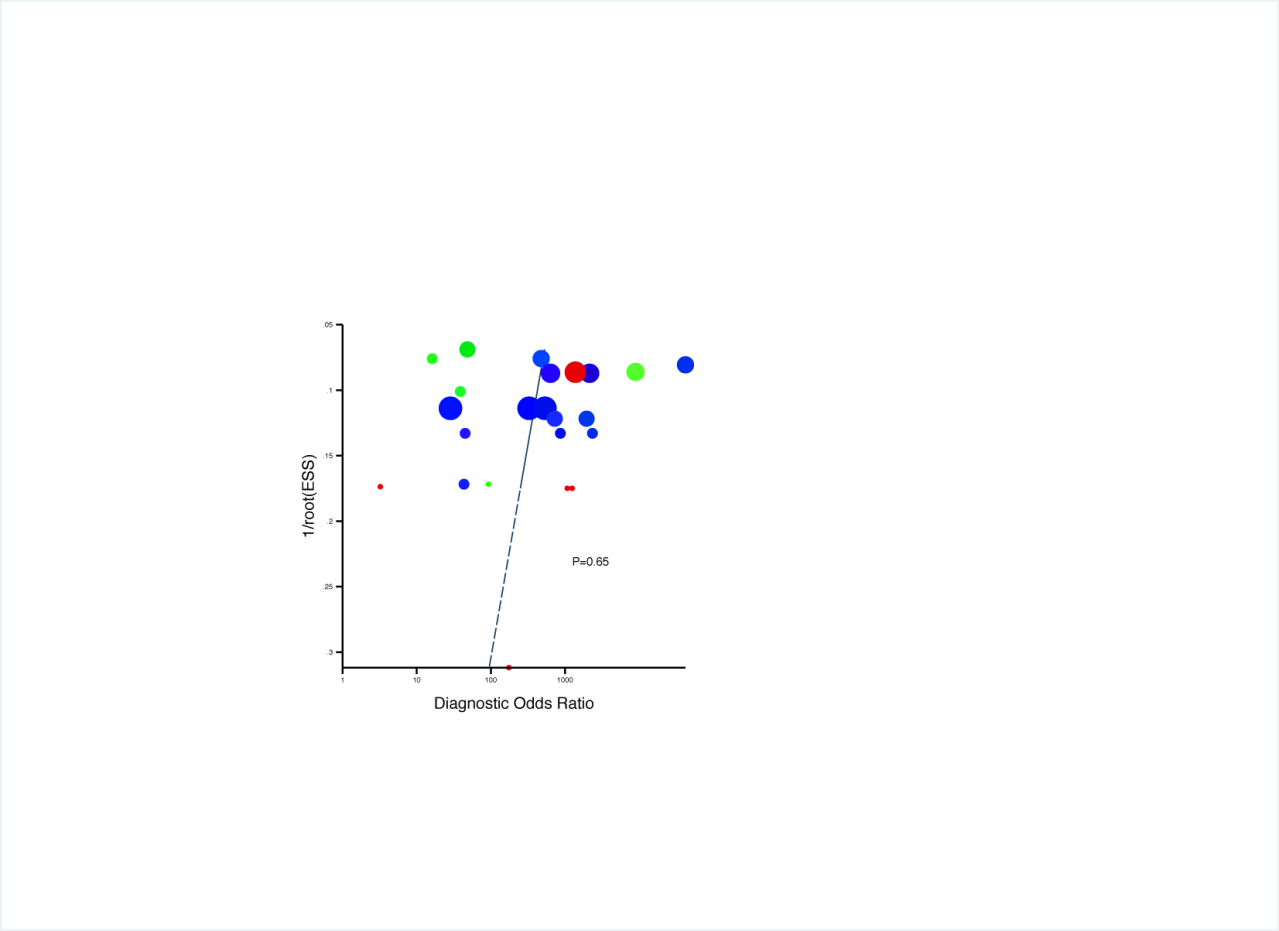
The funnel plots for publication bias. The red solid circle represents for PCR-based RDTMs, blue for ELISA-based RDTMs and green for other RDTMs. The areas of the circles stand for the number of cases. The Deek test was not significant (p = 0.69).

The 26 studies included diagnostic studies enrolling 632 patients infected with T. *marneffei* and 2,612 negative controls between 1996 and 2016 in Thailand, Vietnam and China (Table 1). The methodological quality of the included studies was showed in S1 Fig. There were only 3 studies described the reference test results were interpreted blind to the results of the index test or blinding is dictated by the test order, and the other trails without any blinding method (Table 1). The funnel plots for publication bias (Fig 2) show symmetry and the Deek test was not significant (p = 0.65). These results indicated that there was no potential for publication bias.

### Overall accuracy of rapid diagnosis

**Fig 3:**
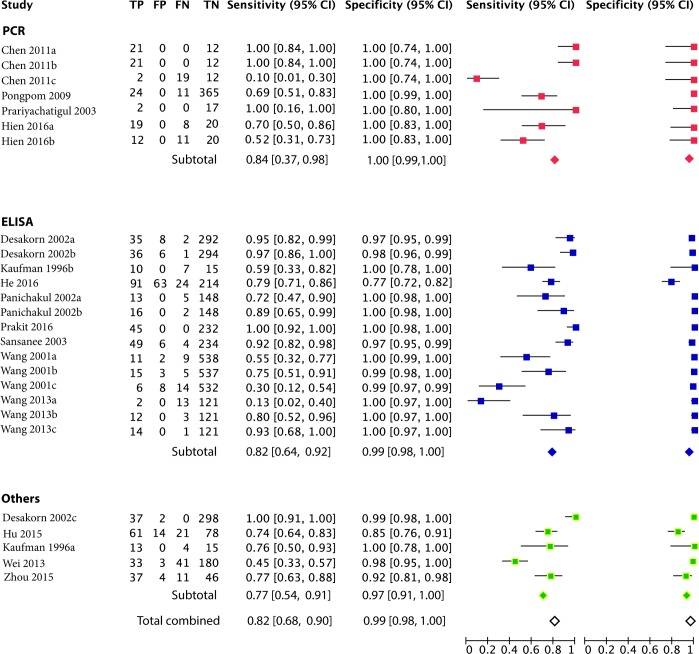
Forest plot of the combined RDTMs of a) sensitivity & specificity. The red box represents for PCR-based RDTMs, blue for ELISA-based RDTMs and green for other RDTMs. a) The hollow rhombus stands for the pooled sensitivity and specificity of entire studies. The rhombus filled with red stands for the pooled sensitivity and specificity of PCR-based RDTMs. The rhombus filled with blue stands for the pooled sensitivity and specificity of ELISA-based RDTMs. And the rhombus filled with green stands for the pooled sensitivity and specificity of other RDTMs.

To determine the sensitivity and specificity of the studies, the authors totally test 5,594 cases or control samples to identify the T. *marneffei* infections. The pooled specificity and sensitivity of the rapid diagnosis assay could be a useful tool for prompt diagnosis. Combined sensitivity, specificity, DLR positive and DLR negative were 0.82 (95% CI: 0.68–0.90), 0.99 (95% CI: 0.98–1.00), 38.92 (95% CI: 19.17–79.00) and 0.24 (95% CI: 0.16–0.36), respectively, after analysing the different types of rapid diagnosis, regardless of the detection methods (Fig 3 and S2 Fig). The SROC curve is well established of summarizing the performance of a diagnostic test among all studies. The area of under SROC was 0.99 (95% CI: 0.98, 1.00) (S3 Fig).

According to the experimental method, the included trials can be divided into three subgroups, the PCR subgroup, the ELISA-based subgroup and the others.

### Molecular diagnosis

Four articles, including seven trials, used the PCR-based experimental technique to detect *T*. *marneffei*. In these studies, 108 patients and 414 controls between 2003 and 2016 in Thailand, China and Vietnam were recruited in the studies. As shown in Fig 3, specificity seemed to be more consistent across the studies than sensitivity, with sensitivity estimates ranging from 0.10 to 1.00 and specificity estimates achieving a stable 1.00. Overall, for all RDTM of PCR, combined sensitivity was 0.84 (95% CI: 0.37–0.98), while combined specificity was 1.00 (95% CI: 0.99–1.00).

### ELISA based serodiagnosis assays

The ELISA-related experimental methods were also employed to detect the fungus. The methods were analysed in eight articles (14 studies) comprising 320 patients with *T*. *marneffei* and 1,873 controls in Thailand and China between 1996 and 2016. A recombinant T. *marneffei* mannoprotein (Mp1p) for the serodiagnosis of T. *marneffei* infection was developed to be a common use ELISA-based antibody RDTM in clinical. Evaluation of this test revealed high specificity (100%) and approximately 80% (95% CI, 52%-96%) sensitivity in HIV seropositive patients infected with T. *marneffei*. Compiled with antibody and antigen tests for the diagnosis of T. *marneffei* infection had a higher sensitivity of 88%, with a positive predictive value of 100% and a negative predictive value of 96% (Table 1). Pooled sensitivity and specificity were 0.82 (95% CI: 0.64–0.92) and 0.99 (0.98–1.00) (Fig 3), respectively.

### Other RDTMs

Along with PCR- and ELISA-related experimental methods, researchers also developed other detection technologies to diagnose *T*. *marneffei*. Five different experimental methods were considered in the meta-analysis, including latex agglutination (LA), G test, spectrophotometric detection and Giemsa staining. Pastorex Aspergillus is a latex agglutination test kit using a monoclonal antibody to detect Aspergillus fumigatus galactomannan in serum specimens from patients with aspergillosis. The reagent was used to detect galactomannan in an experimental infection with T. *marneffei*. However, the titer of antigen detected was lower than that in infection with Aspergillus. The combined sensitivity and specificity were 0.70 (95% CI: 0.64–0.76) and 0.96 (95% CI: 0.95–0.98), respectively (Fig 3).

## Discussion

Our study found that the current RDTM is extremely highly specific and with modest and highly variably sensitivity. In contrast, ELISA-based methods work more rapidly and have been used effectively for specific detection of *T*. *marneffei*. In addition, our meta-analysis revealed that nested PCR was more accurate in detecting *T*. *marneffei* in clinical culture samples or fresh tissues. It can improve the detection accuracy when using certain measures, for instance, blood fungus culture for 3 or 4 days, to increase the DNA extraction yield from white blood cells. However, the ELISA-based RDTM showed high specificity and approximately 80% sensitivity, even in non-culture serum. Until now, rapid diagnosis of *T*. *marneffei* infections has been a significant challenge, although serodiagnostic assays have been reported as powerful tools for detecting pathogenic fungi.

Molecular tools for the detection of the fungus is based on taxon-specific primers designed from the internally transcribed spacer and ribosomal RNA gene for the fungal strains, which enables early and rapid diagnosis [21, 22]. The specificity of *T*. *marneffei* primers was tested in a nested PCR from series case reports. This biology approach was nearly 100% successfully to amplify *T*. *marneffei* DNA and have been developed to identify *T*. *marneffei* from a skin biopsy. Use of new modified PCR-based techniques for the rapid detection of the infection needs to be studied and implemented further. Moreover, most of T. *marneffei* exist in white blood cells, and the authors used liquid nitrogen grinding crushing method to break cell wall, which cannot break the cell wall completely. Thus, the cell wall broken method can only extract a small amount of DNA and reduce the efficiency of PCR amplification seriously. Compared with nested PCR, the detection sensitivity of traditional PCR that the authors used was lower for the lower amplification sensitivity.

The use of monoclonal antibody based sandwich enzyme-linked immunosorbent assay offers advantages for the highly specific and sensitive detection of *T*. *marneffei* antigens in clinical specimens from patients with laboratory confirmed *T*. *marneffei* infection. Whereas the IgM from clone 8C3 responses was immobilized onto the wall of microtiter plates were higher against both yeast and mycelial antigens [47]. The antigen in serum or urine was significantly higher against with biotinylated polyclonal rabbit anti-*T*. *marneffei* antibody. Especially T. *marneffei* often occurs in immune dysfunction, it is easily combined with other opportunistic infection, which would greatly affect the ELISA accuracy in the practical use. Also ELISA procedures the antigen is detected clearly in the early phase whereas the antibody only can detect during the intermediate stage, it would be impact on the different phases. In our study, the positivity rate for antigen was significantly higher for antibody as compared to the early diagnosis. When used in combination with antigen and antibody tests, it improves the detection rate substantially.

Several additional methods for detecting circulating *T*. *marneffei* antigens have been developed with the same polyclonal antibody and compared with the ELISA for the detection of *T*. *marneffei* urinary antigen. A dot blot ELISA and a latex agglutination (LA) test were developed in which urine specimens from 37 patients with culture-proven penicilliosis and 300 controls (52 healthy subjects and 248 hospitalized patients without penicilliosis) were tested. The overall sensitivities of the tests were as follows: dot blot ELISA, 94%; ELISA, 97%; LA test, 100% [31]. However, a potential future application would be to assess responses to antifungal therapy in serial clinical samples testing during treatment and subsequent follow-up without the isolation.

Our studies also had several potential methodological limitations. In particular, only three of the included studies reported blinded assessment of the RDTM assays and the control subjects in most studies were comprised by healthy subjects and hospital patients without *T*. *marneffei*. Although RDTM gives a dichotomous yes/no answer, discordance lines may be due to one or the other factor of false-positive results. It is difficult assess the risk of bias, although we did not find any discordance exists in reported accuracy between whether blinded versus unblinded. Moreover, this review could have been affected by publication bias, although we searched several sources and updated our searches, but we may have missed some eligible studies in south Asia for searched articles only published in Chinese and English. The last but not the least, cost-effectiveness analysis would seem to be an absolute necessity to evaluate whether potential benefits offset the added costs of routine use of RDTM. Nevertheless, further development of these tests need to be concerned, a larger number of specimens from patients with T. *marneffei* and other virulence potential favoring disease need to be tested.

## Supporting information

S1 Checklist PRISMA 2009 checklist. (DOC)

S1 Fig Summary of the risk of bias and applicability concerns. a) Risk of bias and applicability concerns assessment with an overview of the reviewers’ judgment about each separate domain for each included study. b) Summary of the risk of bias and applicability concerns across the included studies as assessed with QUADAS-2 forms.
(TIF)

S2 Fig Forest plot of the combined RDTMs of diagnostic likelihood ratio (DLR) positive & DLR negative. The hollow rhombus stands for the pooled DLR positive and DLR negative of included studies.
(TIF)

S3 Fig Summary reciver operating curve (SROC) of different RDTMs of T. *Marneffei*. The red solid oval represents for PCR-based RDTMs, blue for ELISA-based RDTMs and green for other RDTMs. the size of the ovals means the quantity of cases. The result of SROC indicates the relationship between the true positive rate (TPR) and the false positive rate (FPR) of the test, as the area under curve used to distinguish T. marneffei cases from non-infection varies.
(TIF)
