# Supplementary figures and images for: Accuracy of rapid diagnosis of Talaromyces marneffei: A systematic review and meta-analysis
Source: PLoS One. 2018 Apr 5;13(4):e0195569. doi: 10.1371/journal.pone.0195569 (PMC5886574; doi:10.1371/journal.pone.0195569)

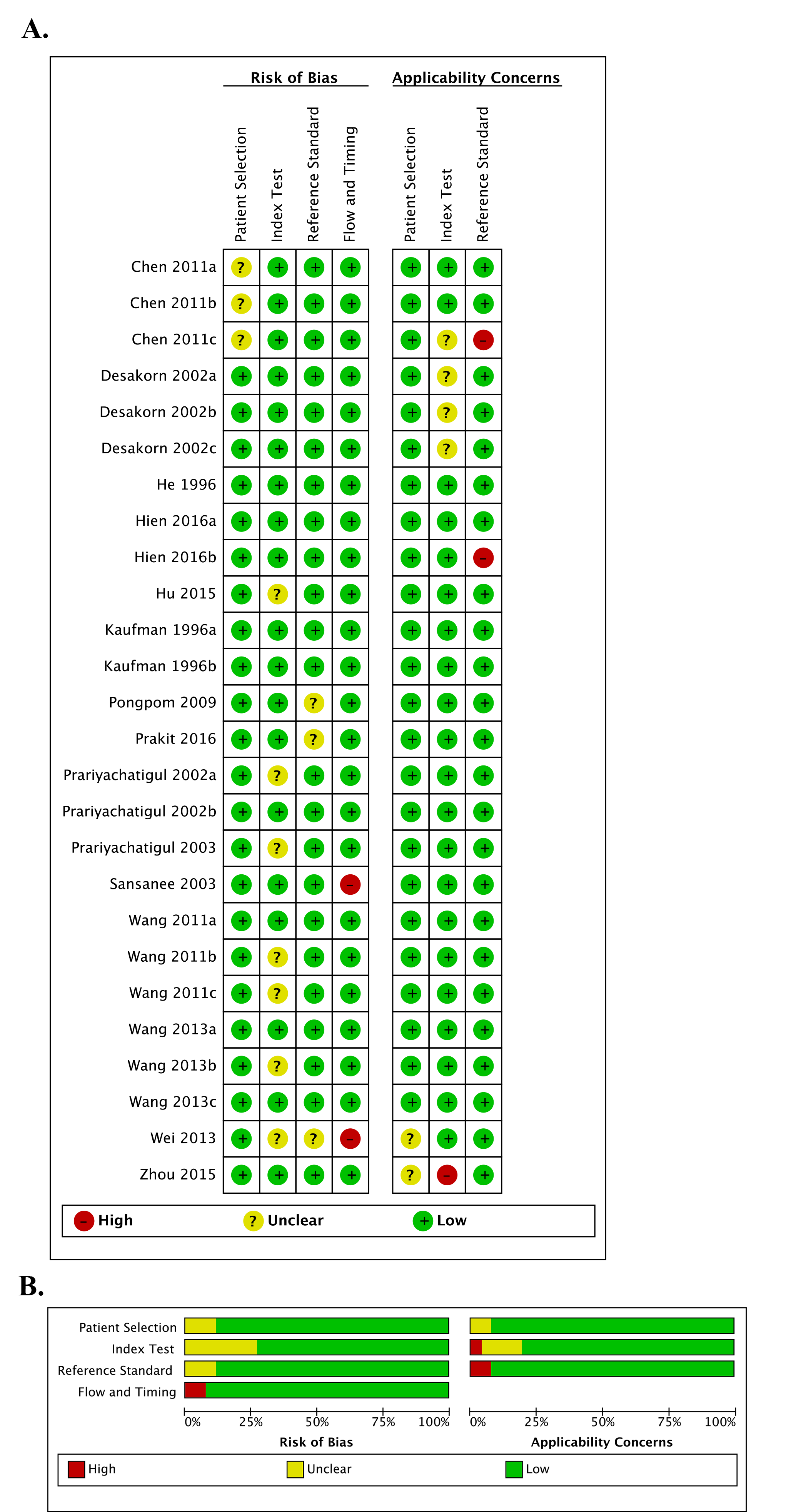

Supplement: S1 Fig — a) Risk of bias and applicability concerns assessment with an overview of the reviewers’ judgment about each separate domain for each included study. b) Summary of the risk of bias and applicability concerns across the included studies as assessed with QUADAS-2 forms. (TIF) [file pone.0195569.s002.tif]

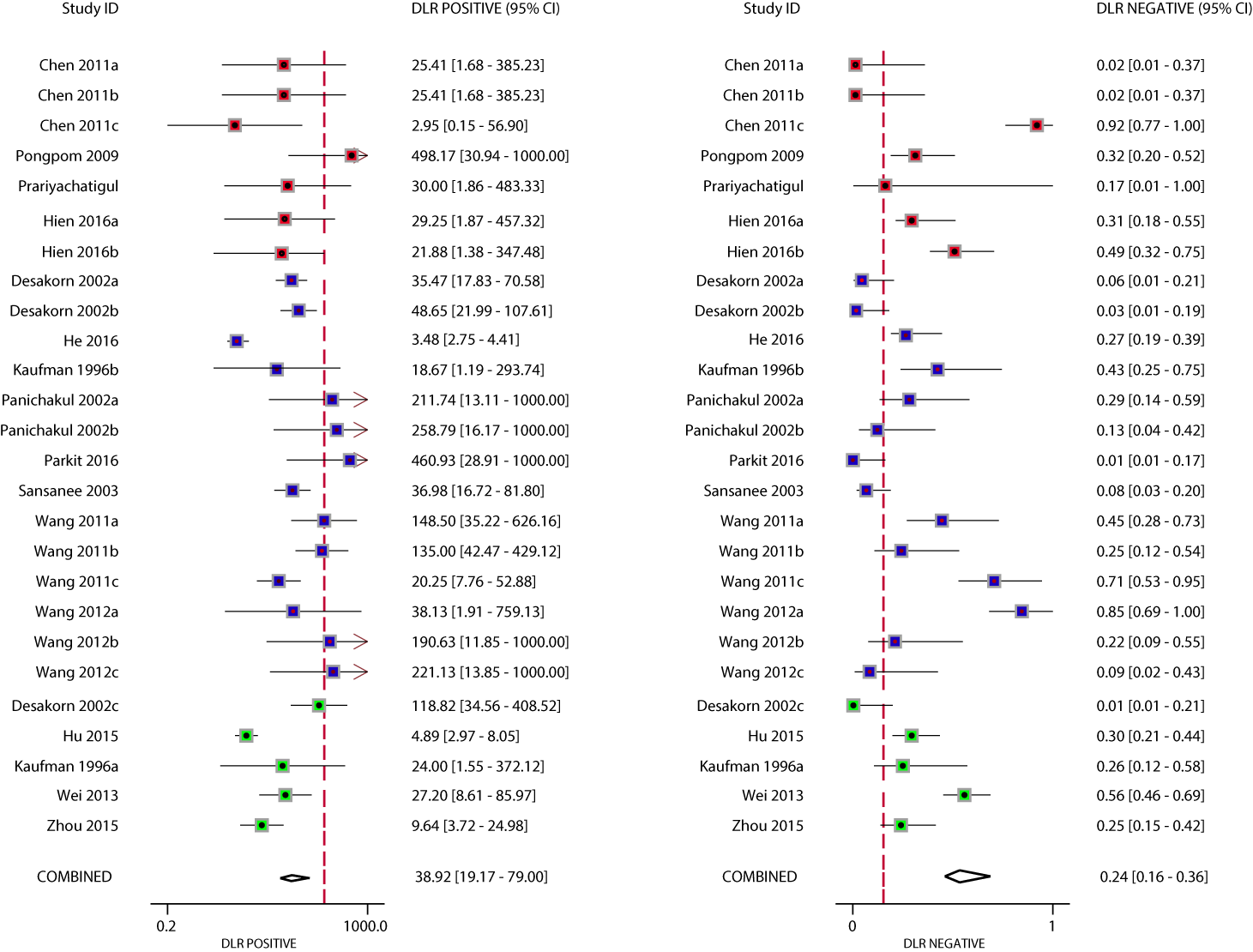

Supplement: S2 Fig — The hollow rhombus stands for the pooled DLR positive and DLR negative of included studies. (TIF) [file pone.0195569.s003.tif]

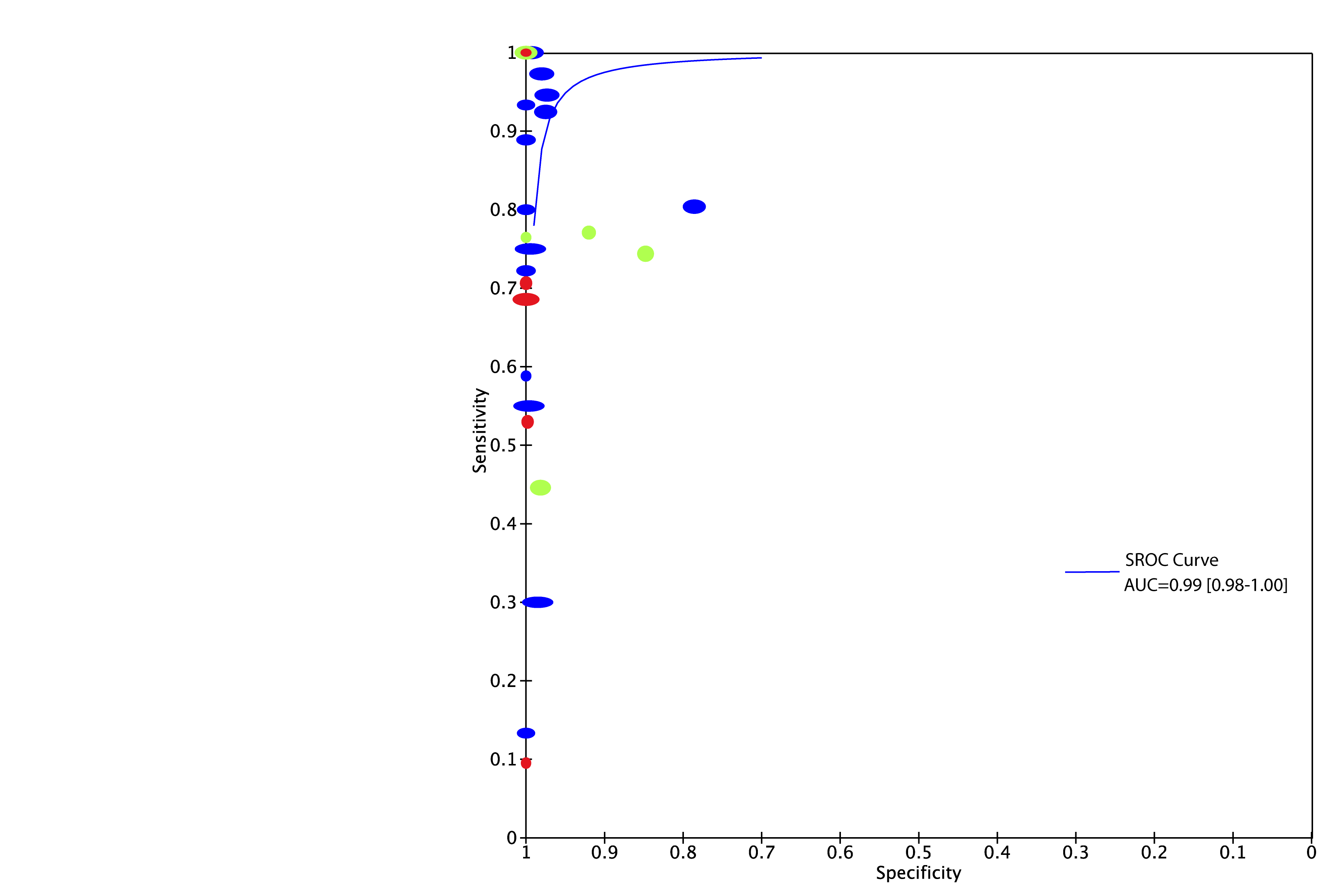

Supplement: S3 Fig — The red solid oval represents for PCR-based RDTMs, blue for ELISA-based RDTMs and green for other RDTMs. the size of the ovals means the quantity of cases. The result of SROC indicates the relationship between the true positive rate (TPR) and the false positive rate (FPR) of the test, as the area under curve used to distinguish T. marneffei cases from non-infection varies. (TIF) [file pone.0195569.s004.tif]
